# Epitope-Based Chicken-Derived Novel Anti-PAD2 Monoclonal Antibodies Inhibit Citrullination

**DOI:** 10.1155/2021/6659960

**Published:** 2021-04-12

**Authors:** Masayoshi Aosasa, Md Saddam Hossain, Tomoko Sakata, Keita Koga, Takanari Shigemitsu, Yuji Shoya, Motonori Yamaguchi, Kenji Saito, Mujo Kim

**Affiliations:** ^1^Pharma Foods International Co. Ltd., 1-49 Goryo-Ohara, Nishikyo Ku, Kyoto 615-8245, Japan; ^2^International PAD Research Center, 1-49 Goryo-Ohara, Nishikyo Ku, Kyoto 615-8245, Japan

## Abstract

The aberrant upregulation of protein arginine deiminase 2- (PAD2-) catalyzed citrullination is reported in various autoimmune diseases (rheumatoid arthritis and multiple sclerosis) and several cancers. Currently, there are no anti-PAD2 monoclonal antibodies (mAbs) that can inhibit the citrullination reaction. Here, an epitope ^341^YLNRGDRWIQDEIEFGY^357^ was examined as an antigenic site of PAD2. Chickens were immunized with this epitope, and the generated mAbs were screened for its reactivity against the full-length PAD2. Enzyme-linked immunosorbent assay revealed that six mAbs, which were screened from the phage display library, crossreacted with mouse PAD2. Kinetic analysis revealed that mAbs are bound to PAD2 in the nanomolar range, which indicated a strong binding. Results of the *in vitro* citrullination inhibition assay revealed that the half-maximal effective concentration values of mAbs for the inhibition of histone or benzoyl-L-arginine ethyl ester citrullination were in the range of 6–75 nM which supports strong inhibition capabilities. Alanine scanning of epitope revealed that the peptide fragment ^344^RGDRWIQDEIEF^355^ was responsible for generating strong antibody responses that inhibit the PAD2-catalyzed citrullination reaction. These antibodies can aid in understanding the extracellular PAD2 function and treating diseases associated with aberrant citrullination.

## 1. Introduction

Citrullination is a type of posttranslational modification that involves the production of citrulline, a noncoding amino acid, through the deimination of arginine. This reaction is catalyzed by the peptidyl-arginine deiminase (PAD) family of enzymes. PADs regulate various cellular processes, including transcriptional regulation of gene expression [1], skin keratinization [2], and the maintenance of myelin sheath insulation [3]. Additionally, citrullination promotes the formation of neutrophil extracellular traps (NETs), a mechanism through which neutrophils capture and eliminate pathogens [4, 5]. In humans, the PAD family comprises five calcium-dependent isozymes (PAD1–4 and 6) [6]. Calcium induces conformational changes and consequently activates the enzyme [7]. Recently, PAD2 and PAD4 isotypes, which are mainly expressed in the immune cells, brain, bone marrow, and joints, have piqued the interest of the scientific community for drug discovery [6, 8].

PADs are cytoplasmic or nuclear proteins that lack the transmembrane regions or secretory signal sequences. Hence, the expression and function of PADs are restricted to the cytoplasm [9]. However, recent studies have demonstrated that the expression of PADs is upregulated during inflammation, which results in the upregulation of citrullination, the activation of NETosis, and, consequently, the release of PADs from the damaged cells [10, 11]. Extracellular PADs led to excessive citrullination of proteins, and aberrantly upregulated citrullination are reported in several autoimmune and inflammatory diseases [12], especially rheumatoid arthritis (RA). In the synovial fluid (SF) of patients with RA, more than 100 citrullinated proteins have been identified, including several neutrophil-associated intracellular proteins, extracellular matrix proteins, and serum proteins, such as albumin, fibrinogen, and immunoglobulin [13, 14]. Hence, deiminated proteins function as neoantigens and promote the production of anti-citrullinated protein antibodies (ACPAs), which mediate the local inflammatory response and exacerbate the severity of RA [15]. ACPAs are found in approximately 70% of patients with RA. Additionally, the activity of PADs is detected in the SF of patients with RA [16, 17]. Spengler et al. [10] detected both PAD2 and PAD4 proteins in the cell-free SF of patients with RA although their expression levels varied in different patients. Interestingly, an *ex vivo* study by Zhou et al. [18] reported that live neutrophils can inherently express catalytically active PAD4 on the cell surface and that active PAD2 is released spontaneously into the culture media from neutrophils without stimulation.

In addition to its involvement in RA progression, PAD2 is involved in the onset and progression of multiple sclerosis (MS) [19], endotoxin-induced lethality [20], and breast cancer [21]. Currently, there are no specific drugs to inhibit PAD2. The roles of intracellular or extracellular citrullination in the initiation or progression of RA pathogenesis are unclear. Therefore, the inhibition of extracellular PAD2 using a specific monoclonal antibody (mAb) will aid the elucidation of the biological roles of this isozyme and the treatment of specific diseases associated with dysregulated PAD2 activity. In this study, we aimed to develop a novel PAD2-specific mAb that could inhibit the citrullination activity of PAD2.

## 2. Materials and Methods

### 2.1. Materials

Keyhole limpet hemocyanin- (KLH-) modified peptide antigen (epitope) was purchased from SCRUM Inc. (Tokyo, Japan). Freund's adjuvant (complete or incomplete) was procured from WAKO Pure Chemical Industries, Ltd. (Osaka, Japan). The RNA isolation reagent TRIzol and the high pure RNA isolation kit were purchased from Life Technologies (California, USA) and Roche Diagnostics (Basel, Switzerland), respectively. Ficoll-Paque PLUS was purchased from Cytiva (Marlborough, USA). The reagents used in the DNA manipulation procedures were purchased from Takara (Kusatsu, Japan). XL1-Blue electrocompetent cells and VCS-M13 were procured from Stratagene (California, USA). The H-chain or L-chain expression vector pcDNA3.4 was obtained from Invitrogen (California, USA). The Expi293 expression system was purchased from Thermo Fisher Scientific (Waltham, USA). Restriction enzymes were purchased from New England Biolabs (Massachusetts, USA). The oligonucleotides for cloning and the DNA sequences of recombinants were obtained from Eurofins Genomics (Tokyo, Japan). Recombinant human PAD2 (rhPAD2) was purchased from Cayman Chemical (Michigan, USA) and was used for all experiments unless otherwise stated. Substrate N*α*-benzoyl-L-arginine ethyl ester (BAEE), and histone from calf thymus were from Peptide Institute Inc. (Ibaraki, Japan) and Sigma-Aldrich (Missouri, USA), respectively. Antibody purification column protein A was purchased from ProteNova (Kagawa, Japan).

### 2.2. Screening of Epitopes

In order to incite strong antigenicity in chicken through immunization, a high-throughput screening of epitopes from PAD was performed. Previously, we and our group succeeded in developing epitope-based anti-PAD4 antibodies based on proprietary technology Avian lead antibody gene (ALAgene®—a screening and generation platform for developing chicken-derived monoclonal antibody) [22]. PAD2 and PAD4 have identical properties both in structure and function [23]. Therefore, we selected the immunogenic epitope of PAD2, positioned almost the same as the previously published PAD4 epitope [22]. Subsequently, protein-protein BLAST (Blastp) [24] analysis was performed to examine the percentage similarity between the screened epitope and the chicken PAD2 protein sequence.

### 2.3. Immunization of Chicken and RNA Isolation

Three-month-old Boris Brown chickens were intraperitoneally immunized once every 4 weeks with 333 *μ*g of KLH-modified epitope of PAD2 emulsified in Freund's complete or incomplete adjuvant. The blood sample was collected from the wing vein every other week. The antibody titers were measured using enzyme-linked immunosorbent assay (ELISA). At the final immunization, the antigen diluted in phosphate-buffered saline (PBS) was injected intravenously. At day 3 postfinal immunization, the spleen of the chicken was collected. Lymphocytes were isolated using density-gradient centrifugation with Ficoll-Paque PLUS. RNA was extracted from the lymphocytes using TRIzol and purified using the RNA isolation kit.

### 2.4. Phage Library Preparation, Panning, and Expression of Anti-PAD2 Antibodies

A previously published protocol was used for the preparation of chicken-derived chimeric anti-PAD2 antibody [25]. Briefly, the extracted RNA was subjected to reverse transcription to synthesize complementary DNA. The variable regions (heavy chain (V_H_) and light chain (V_L_)) of the antibodies were amplified using PCR. The purified V_H_ and V_L_ fragments were assembled using a single-chain variable fragment (scFv) linker. The linker is rich in glycine for flexibility and is composed of 15 amino acids (GGGGSGGGGSGGGGS). The scFv fragments were then ligated with the phagemid vector pPDS (accession number D50401). This recombinant vector was electroporated into the XL1-Blue Electrocompetent Cells. The culture was infected with VCS-M13 in a medium containing ampicillin (50 *μ*g/mL), tetracycline (25 *μ*g/mL), kanamycin (25 *μ*g/mL), and isopropyl *β*-D-1-thiogalactopyranoside (0.1 mM) overnight. The cells were harvested using centrifugation. The prepared scFv library-containing supernatant was filtered, precipitated with polyethylene glycol, and stored at 4°C for panning.

For each cycle of panning, the plate was coated with full-length rhPAD2 (5 *μ*g/mL) in PBS. The PAD2-specific antibody clones were enriched through five cycles of panning. The library exhibiting increased reactivity was subjected to phage screening. Single-phage fragment antibody clones were cultured in a 96-well plate for phage production. The resulting phages were screened for specific binding to PAD2 using ELISA. The clones that bound to PAD2 but not to bovine serum albumin (BSA) were scored as PAD2 specific.

The resulting positive clones were subjected to sequencing. scFvs with distinct V_H_ and V_L_ sequences were regarded as separate clones. The DNA sequence encoding scFv antibody was PCR amplified to obtain the genes encoding an H chain variable region and an L chain variable region of the chicken antibody. The H chain and L chain variable region sequences were cloned into the mouse/chicken chimeric antibody (IgG) expression vector pcDNA3.4. Paired heavy and light chain expression vectors were transfected into the Expi293 expression system, following the manufacturer's instructions. After 5 days of culturing, fully assembled IgG antibodies were purified from the culture supernatant using protein A column chromatography. The purity of IgG was evaluated using sodium dodecyl sulfate-polyacrylamide gel electrophoresis (SDS-PAGE). The concentration of IgG was determined spectrophotometrically at 280 nm. The purified chimeric IgG was preserved and used for further experiments.

### 2.5. ELISA for Evaluating the Reactivity of Chimeric IgG

To determine the chimeric IgG reactivity, rhPAD2, recombinant mouse PAD2 (rmPAD2), or BSA (control antigen) was used. rhPAD2 or rmPAD2 was expressed in the Expi293 expression system separately as described previously [26, 27]. Briefly, the 293F cell line was transfected with pcDNA3.4 containing a PAD2-specific clone. After three days of culture, the cells were harvested to isolate PAD2, which was used for further experiments. rhPAD2 or rmPAD2 5 *μ*g/mL in PBS was coated onto a Maxisorp Nunc-Immuno™ ELISA plate (NUNC, USA) overnight at 4°C. The plate was then blocked with 25% BlockAce in PBS at 37°C for 1 h. Next, the plate was washed with PBS containing 0.1% Tween 20. Fourfold serially diluted chimeric IgG (starting from 200 ng/mL) was added to the wells and the plate was incubated for 1 h at 37°C. After washing the plate, goat anti-mouse IgG (H+L) horseradish peroxidase-conjugated secondary antibody (KPL; 1 : 1000) was added to the wells and the plate was incubated for 1 h at 37°C. The plate was washed again and 3,3′,5,5′-tetramethylbenzidine microwell peroxidase substrate system was added to the wells to develop the color. Color intensity was quantified by measuring the absorbance at 450/650 nm using an EMax Plus spectrophotometer (Molecular Devices, California, USA). The half-maximal effective concentration (EC_50_) of the chimeric IgG affinity was estimated and calculated using the SoftMax Pro software (Molecular Devices, California, USA).

### 2.6. Affinity Measurement Using Surface Plasmon Resonance (SPR)

The kinetics of binding of chimeric antibodies to PAD2 were assessed using SPR with a Biacore T200 instrument (Cytiva, Marlborough, USA). A mouse antibody capturing kit was used for Biacore Series S Sensor Chip CM5 surface preparation, following the manufacturer's instructions. Briefly, the rabbit anti-mouse polyclonal antibody was immobilized through amine coupling to the free carboxyl groups on the CM5 chip surface using standard NHS/EDC procedures. Next, monoclonal antibodies against PAD2 (2 *μ*g/mL) in HBS-EP buffer (10 mM HEPES; pH 7.4, 150 mM NaCl, 3 mM EDTA, and 0.005% (*v*/*v*) surfactant P20) were captured by the rabbit anti-mouse polyclonal antibody. Further, various concentrations of human PAD2 (0.625, 1.25, 2.5, 5, and 10 *μ*g/mL) in HBS-EP buffer were analyzed using Biacore T200 to generate a kinetic sensorgram. The kinetic parameters were obtained by globally fitting the data to a 1 : 1 binding model using the evaluation curve fitting software. The dissociation rate constant (kd) was determined by fitting the change in the binding response during the dissociation phase, while the association rate constant (ka) was determined by fitting PAD2 binding at different concentrations. The equilibrium dissociation constant (KD) was calculated from the ratio of kd to ka.

### 2.7. Citrullination Assay

An *in vitro* inhibition assay was performed to evaluate the ability of chimeric IgG to inhibit the citrullination activity of rhPAD2. BAEE and histone were used as PAD2 substrates. The generation of citrulline was detected based on a colorimetric measurement [22]. Anti-dinitrophenyl (DNP) antibody was used for negative control [28]. For standard inhibition reactions, 5 nM rhPAD2 was preincubated with fourfold serially diluted (starting from 600 nM) chimeric IgG in 44 *μ*L of reaction buffer (20 mM Tris-HCl (pH 7.6), 1 mM dithiothreitol, and 150 mM NaCl) for 30 min at 25°C. The reactions were initiated in 50 *μ*L aliquots with the addition of the substrate (10 mM) and CaCl_2_ (1 mM). The samples were incubated at 37°C for 4 h and the reactions were quenched using 12.5 *μ*L of 5 M perchloric acid. Next, 40 *μ*L of the reaction mixture was assayed for citrulline activity after mixing it with 100 *μ*L reagent A (0.04% *w*/*v* ferric chloride, 25% *v*/*v* H_2_SO_4_, and 20% *v*/*v* H_3_PO_4_), 50 *μ*L reagent B (0.5% *w*/*v* 2,3-butanedione oxime and 0.01% *w*/*v* thiosemicarbazide), and 10 *μ*L distilled water. The mixture was then boiled for 5 min and cooled to 25°C in an ice bath. The absorbance of the samples was measured at 492 nm. The efficacy of chimeric IgG to inhibit rhPAD2 citrullination activity was normalized with that of anti-DNP antibody.

### 2.8. Epitope Mapping by Alanine Scanning

To elucidate the critical residues within the epitope involved in antibody recognition, epitope mapping was performed. Although there are different methodologies, alanine scanning based on synthetic peptides is one of the most common methods to map the epitope specificity to an antibody molecule [29–31]. In this study, six mutant epitopes were synthesized in which three or two amino acids within the epitope sequences were replaced with alanine (Supplementary file, Table S1) using the GenScript (Tokyo, Japan) with a purity of more than 95%. The dose-dependent reactivity of each epitope mimic with the anti-PAD2 antibodies was evaluated using ELISA as described in an earlier section.

## 3. Results

### 3.1. Screening of Epitope

**Figure 1:**
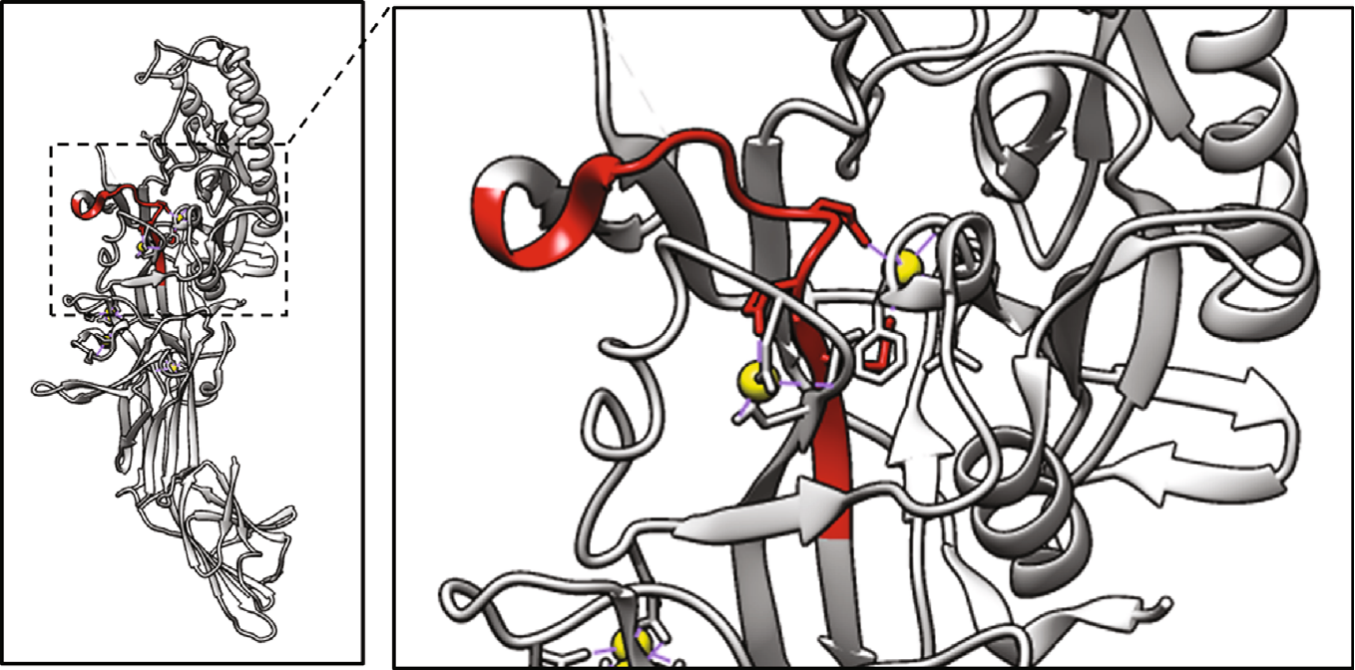
PAD2 ribbon structure (PDB ID: 4N2C). The epitope 341-YLNRGDRWIQDEIEFGY-357 is shown in red, while calcium is shown in yellow. The three-dimensional structure of PAD2 was edited and prepared using UCSF chimera as described previously [32].

The epitope, ^341^YLNRGDRWIQDEIEFGY^357^, was identified (Figure 1). Blastp analysis of this epitope sequence with the chicken protein sequence revealed that the epitope exhibited marked similarity with chicken PAD2 with only two varying residues (data not shown).

### 3.2. Selection of Anti-PAD2 Antibodies from a Phage Display Library

**Figure 2:**
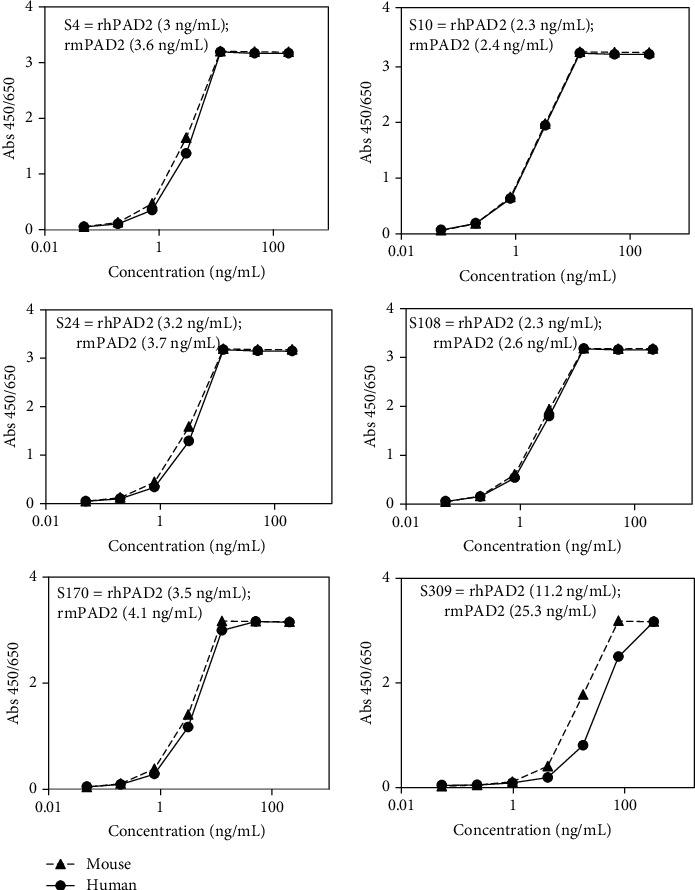
Crossreactivity of chimeric anti-PAD2 antibodies with recombinant mouse PAD2. The values in the bracket indicates EC_50_ values of antibodies against rhPAD2 and rmPAD2 enzymes. The enzyme-linked immunosorbent assay protocol is described in Materials and Methods.

A phage scFv display library constructed using chicken immunized with a selective epitope of PAD2 was panned against rhPAD2 for five cycles. The phage scFv antibody clones specific to rhPAD2 were identified using phage ELISA. Sequence analysis of the V_H_ and V_L_ chains revealed six unique clones specific to S4, S10, S24, S108, S170, and S309. These positive clones were subsequently expressed in the Expi293 system and purified by protein A column chromatography. The extent of purification was confirmed by SDS-PAGE and data showed the clear and well purified band (Supplementary file, Figure S1). The ability of each chimeric IgG to bind to rhPAD2 or rmPAD2 was confirmed using ELISA (Figure 2), and the EC_50_ value of each chimeric IgG was calculated. The binding affinity was inversely proportional to the magnitude of the EC_50_ value. All screened antibodies exhibited crossreactivity with rmPAD2.

### 3.3. Evaluation of Affinity of Antibodies with rhPAD2

The interaction between chimeric antibodies and immobilized rhPAD2 was measured using SPR from a sensorgram. The results of the SPR analysis confirmed that rhPAD2 binds to the antibody with a high affinity. The kinetic constants, including the KD, ka, and kd values, were determined for each mAb and are summarized in Table 1. The ka and kd values were similar for S10 and S170 mAbs, which exhibited KD values of approximately 7.8 and 6.33 nM, respectively. The dissociation values were high for the mAbs S4 and S309, which exhibited KD values of approximately 26 and 42 nM, respectively. However, the KD values of the mAbs S24 and S108 were approximately 80 and 74 nM, respectively.

### 3.4. Citrullination Inhibition by Anti-PAD2 Antibodies

**Figure 3:**
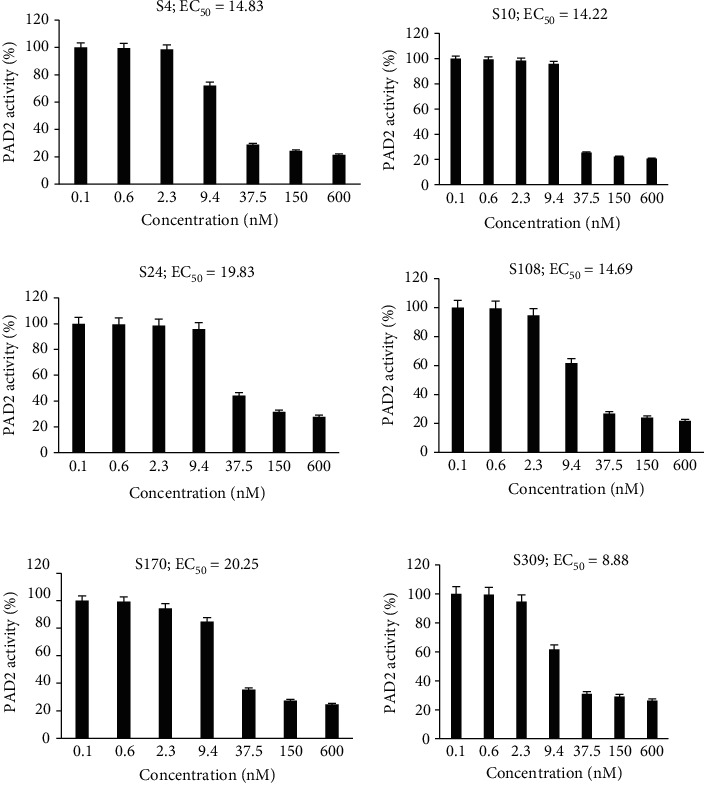
Ability of anti-PAD2 antibodies to inhibit PAD2-mediated citrullination of N*α*-benzoyl-L-arginine ethyl ester (BAEE). S4, S10, S24, S108, S170, and S309 antibodies were incubated with recombinant human PAD2 (rhPAD2) and buffer (20 mM Tris-HCl (pH 7.6), 150 mM NaCl, and 1 mM dithiothreitol) in a total volume of 44 *μ*L for 30 min at 25°C. BAEE and CaCl_2_ (volume: 6 *μ*L) were added simultaneously and the mixture was stirred well. The mixture comprised 5 nM rhPAD2, 10 mM BAEE, 1 mM CaCl_2_, and various concentrations of antibodies (0.1–600 nM). The mixture was incubated at 37°C for 4 h, and 12.5 *μ*L of 5 M perchloric acid was added to stop the reaction. The level of citrullinated BAEE was determined using a colorimetric assay system.

**Figure 4:**
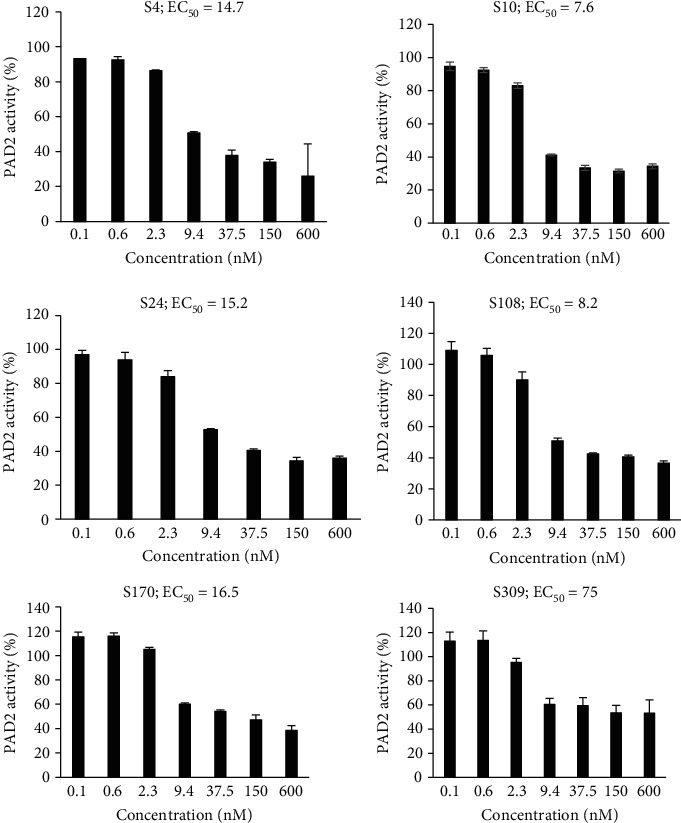
Ability of anti-PAD2 antibodies to inhibit recombinant human PAD2- (rhPAD2-) mediated histone citrullination. The anti-PAD2 antibodies were mixed with rhPAD2 and buffer as described in the legend of Figure 3 at 25°C for 30 min. Histone (1.8 mg/mL) and CaCl_2_ (1 mM) were added simultaneously and the mixture was incubated for 4 h at 37°C. Citrullinated histone was determined using a colorimetric assay.

The effect of anti-PAD2 antibodies on the rhPAD2-mediated citrullination of BAEE and histone from calf thymus was examined. Additionally, the EC_50_ values of the antibodies were measured. The antibodies decreased the citrullination activity of rhPAD2. The antibody-mediated inhibition of citrullination reaction varied with the substrate. For example, the EC_50_ values of the antibodies for the inhibition of BAEE citrullination were in the range of 8.0–20 nM (Figure 3). In contrast, the EC_50_ values of the antibodies for the inhibition of histone citrullination were in the range of 7.0–75 nM (Figure 4). Among the screened antibodies, S10 exhibited the lowest EC_50_ value (approximately 7.6 nM) for the inhibition of histone citrullination. S10 also exhibited the lowest KD values. Other mAb candidates, such as S108 and S4, had EC_50_ values less than 15 nM for the inhibition of histone citrullination.

### 3.5. Epitope Mapping by Alanine Scanning

The epitopes substituted with alanine were assayed for reactivity with chimeric antibodies using ELISA. The EC_50_ of the epitope, in which the first three amino acids (^341^Y–^343^N) were replaced with alanine, was 1.5-fold higher than that of the native epitope (Table 2). A similar change in EC_50_ value was observed upon substitution of the last two amino acids of the epitope with alanine. However, the EC_50_ value of the epitope in which the next three amino acids (^344^R–^346^D) were substituted with alanine increased by 3-fold to 250-fold when compared to that of the native epitope. Moreover, substituting the next three amino acids (^347^R–^349^I) with alanine significantly altered the binding affinity of antibodies and markedly increased the EC_50_. Additionally, the substitution of ^350^Q–F^355^ with alanine markedly increased the EC_50_ in some mAb candidates, such as S4, S108, S170, and S309, which lost their binding affinity completely. This indicated the crucial role of these residues for mAb binding. A typical epitope is approximately 8–10 amino acids in length. Thus, the screened mAbs may bind within the ^344^RGDRWIQDEIEF^355^ fragment of the epitope.

## 4. Discussion

As humans and mice are phylogenetically related, they have various highly conserved proteins. Hence, the immunization of mice with human proteins sometimes leads to thymic tolerances [33] and elicits weak antibody responses. Alternatively, knockout mice can be generated, which is laborious and time-consuming and can often disrupt tolerance [34]. In contrast, chicken is an alternative host for immunization as it elicits immune responses against epitopes that are mostly conserved in multiple orthologs of a mammalian protein [35]. Moreover, the immunoglobulin repertoire of chicken is suited for antibody phage display. Chickens generate immunoglobulin from a single set of V_H_ and V_L_ germ-line sequences [36]. The entire chicken antibody repertoire can be readily analyzed using only a set of four PCR primers [37]. Previously, Nakamura et al. demonstrated the advantage of using chicken as an immune host against a highly conserved mammalian prion protein and obtained a vast repertoire of the scFv library [25]. Interestingly, human PAD2 has 93% and 68% sequence similarities with mouse PAD2 and chicken PAD2, respectively. These findings suggested that chicken is an optimal host for immunization. In this study, the selected epitope exhibited 88% sequence similarity with chicken PAD2 (data not shown), which indicated that adequate immune responses cannot be elicited even in chicken. However, the serum titer in the immunized chicken was higher than 64000 by dilution factor, which is by our expertise is sufficient for the successful preparation of a phage library.

To characterize the specificity of mAb binding, alanine scanning of the epitope was performed. The results of ELISA revealed that the peptide fragment, ^344^RGDRWIQDEIEF^355^, was crucial for the binding of mAbs (Table 2). Previously, anti-PAD4 antibody also binds with the positions ^340^E-^356^Y of PAD4. We hypothesized that this peptide fragment has an importance for the inhibition of citrullination activity of both PAD2 and PAD4. Details of crystallography analysis for antibody binding with this region are in progress; we hope that in next publication, we can put sufficient information.

Generally, PADs are inactive under physiological conditions of cells [38]. Exposure to toxins, infections, or genetic mutations may promote apoptosis and necrosis in the cells. Consequently, PADs are activated in dying cells due to the influx of calcium ions. The efficiency of citrullination by PAD2 and PAD4 varies depending on the substrates [39]. PAD2 can citrullinate plasma protein fibrinogen more efficiently than PAD4. However, PAD4 can citrullinate histone H3 more efficiently than PAD2 [40]. These findings suggest that both isoforms exhibit variable enzymatic activity [23]. Autoimmune diseases are characterized by extracellular citrullination, especially extracellular hypercitrullination [12]. The anti-PAD2 antibody reported in this study may directly inhibit the catalytic activity of PAD2, which inhibits the interaction of PAD2 with the substrates. Alternatively, the anti-PAD2 antibody may impede calcium binding and dimerization, which are critical for PAD2 activity. The anti-PAD2 antibody will prevent extracellular citrullination rather than intracellular citrullination, which aids in preserving the innate ability of cells to citrullinate the intracellular substrate of PAD2. It can be supposed that a drug containing an anti-PAD2 antibody will have lesser side effects than a small molecule inhibitor of PAD2, which is important for patient safety and compliance. Although, the conserved amino acid sequence among PAD isoforms is in the range of 50–55%, however, the mAbs generated in this study specifically recognize the PAD2 isoform and not the other PAD enzyme isoforms (data not shown). This is in contrast to the properties of the small molecule inhibitor of PAD, which simultaneously inhibit citrullination catalyzed by PAD1, PAD2, PAD3, and PAD4 [8, 41]. Additionally, we are expecting that the anti-PAD2 antibody can do clearance of extracellular PAD2 by targeting the complement activating immune complexes or Fc receptor-mediated endocytosis by phagocytic cells [42].

The characteristics of PAD2, which is expressed in the brain, involve the deregulation of citrullination by promoting the damage of fatty myelin sheaths around the axons of the brain and spinal cord [19]. Hence, the inhibition of PAD2 through the anti-PAD2 antibody can be an effective therapeutic strategy for MS. The inhibition of PAD2 using small molecule inhibitors may not be effective for MS as they can block intracellular citrullination, which is essential for the production of functional myelin. Some studies have reported that the blood-brain barrier (BBB) is damaged in MS [43]. Thus, an antibody-based drug may cross the BBB.

## 5. Conclusion

Small molecule inhibitors of PAD have yielded promising results in the mouse model [44, 45]. However, the design of clinical trials involving these small molecule inhibitors has been criticized as inhibitors suppress innate immunity and consequently increase the susceptibility of patients to infections [46]. Conventional mAb treatments for RA target cytokines, such as TNF-*α*, IL-1-*β*, or IL-6. However, the neutralization of these cytokines can lead to the development of serious side effects. ACPA can be detected years before the onset of clinical disease. Thus, antibody-mediated inhibition of PAD2 activity can be a novel therapeutic approach for diseases associated with aberrant citrullination.

## Figures and Tables

**Table 1:** Kinetics of binding of chimeric anti-PAD2 monoclonal antibodies (mAbs) with recombinant human PAD2.

Anti-PAD2 mAb	ka (1/Ms)	kd (1/s)	KD (M)
S4	2.90*E* + 04	1.0*E* − 03	2.63*E* − 08
S10	3.19*E* + 04	2.49*E* − 04	7.80*E* − 09
S24	7.01*E* + 03	5.65*E* − 04	8.07*E* − 08
S108	7.18*E* + 03	5.29*E* − 04	7.36*E* − 08
S170	1.64*E* + 04	1.04*E* − 04	6.33*E* − 09
S309	2.54*E* + 04	1.08*E* − 03	4.27*E* − 08

**Table 2:** Alanine scanning of epitopes along with their half-maximal effective concentration. EC_50_ (ng/mL) values determined using enzyme-linked immunosorbent assay^∗^.

Residue no.	341	342	343	344	345	346	347	348	349	350	351	352	353	354	355	356	357	EC_50_, original epitope
Amino acid	Y	L	N	R	G	D	R	W	I	Q	D	E	I	E	F	G	Y	
Anti-PAD2 mAb S4	6.12			**42.72**			**1.68 × 10** ^**7**^			**N.D.**			**105.90**			9.17		4.45
Anti-PAD2 mAb S10	6.18			**32.82**			**659.60**			**4.25 × 10** ^**5**^			**1.64 × 10** ^**12**^			9.08		4.49
Anti-PAD2 mAb S24	7.15			**123.90**			**1550.00**			**119.70**			**250.70**			9.16		5.08
Anti-PAD2 mAb S108	4.93			**12.56**			**50.09**			**N.D.**			**1.30 × 10** ^**5**^			5.76		4.16
Anti-PAD2 mAb S170	3.01			**553.30**			**1.24 × 10** ^**7**^			**6.24 × 10** ^**5**^			**N.D.**			4.35		2.15
Anti-PAD2 mAb S309	19.25			**295.40**			**332.60**			**N.D.**			**N.D.**			**31.90**		11.53

^∗^Bold region showed no significant reactivity in alanine scanning.

## Data Availability

The data used to support the findings of this study are available from the corresponding author upon request.

## Supplementary Materials

Supplementary Materials Table S1: list of mutant epitopes for alanine scanning. Figure S1: the level of purification of PAD2 antibodies in SDS-PAGE.
