# Supplementary material for: Epitope-Based Chicken-Derived Novel Anti-PAD2 Monoclonal Antibodies Inhibit Citrullination
Source: J Immunol Res. 2021 Apr 12;2021:6659960. doi: 10.1155/2021/6659960 (PMC8055403; doi:10.1155/2021/6659960)
Supplement: Supplementary Materials — Table S1: list of mutant epitopes for alanine scanning. Figure S1: the level of purification of PAD2 antibodies in SDS-PAGE. [file 6659960.f1.docx]

# Supplementary Material

# Epitope-based chicken-derived novel anti-PAD2 monoclonal antibodies inhibit citrullination

Masayoshi Aosasa^1, 2^; Md Saddam Hossain^1, 2^; Tomoko Sakata^1, 2^; Keita Koga^1^; Takanari Shigemitsu^1, 2^; Yuji Shoya^1, 2^; Motonori Yamaguchi^1^; Kenji Saito^1, 2,*^ and Mujo Kim^1^

^1^Pharma Foods International Co., Ltd, 1-49 Goryo-Ohara, Nishikyo Ku, Kyoto 615-8245, Japan.

^2^International PAD Research Center, 1-49 Goryo-Ohara, Nishikyo Ku, Kyoto 615-8245, Japan.

Table S1: List of mutant epitopes for alanine scanning.

| Seq ID No | Mutant epitopes* |
| --- | --- |
| 1. | **AAA**RGDRWIQDEIEFGY |
| 2 | YLN**AAA**RWIQDEIEFGY |
| 3 | YLNRGD**AAA**QDEIEFGY |
| 4 | YLNRGDRWI**AAA**IEFGY |
| 5 | YLNRGDRWIQDE**AAA**GY |
| 6 | YLNRGDRWIQDEIEF**AA** |

*Alanine substitutions are shown in bold font. The purity was confirmed using high-performance liquid chromatography-tandem mass spectrometry (>95%)

Figure S1: The level of purification of PAD2 antibodies in SDS-PAGE. Equal amounts of 0.5 ug of antibodies were analyzed by SDS-PAGE under reducing conditions (5%–20% gradient gel). Two bands of around 50 and 25 kDa, corresponding to the heavy and light chains were detected.

S4

S10

S24

S108

S170

S309

**kDa**

　
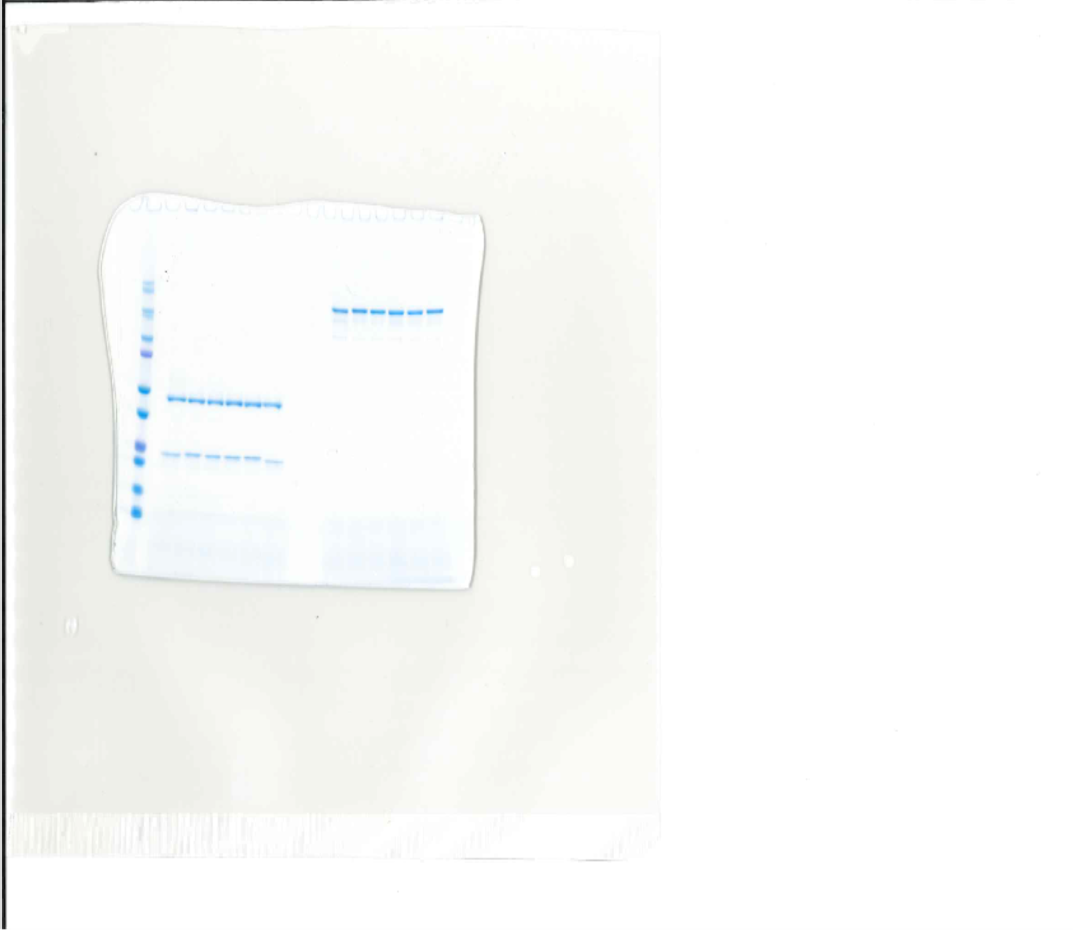


**50-**

**15-**

**~25 kDa**

**~50 kDa**

**25-**

**150-**

**75-**
